# Supplementary material for: RSim: A reference-based normalization method via rank similarity
Source: PLoS Comput Biol. 2023 Sep 1;19(9):e1011447. doi: 10.1371/journal.pcbi.1011447 (PMC10501661; doi:10.1371/journal.pcbi.1011447)
Supplement: S1 Text — Descriptions of how the datasets were generated in simulations. (PDF) [file pcbi.1011447.s001.pdf]

Supplement Material

Numerical Experiment Setup

Summary of notations We denote sample size by n, and the number of taxa by d. $\mathcal{J}_1$ represents the set of differential abundant taxa, and $\mathcal{J}_0$ represents the rest of the taxa. $X_{i,j}$ is the raw count from the data set. $A_{i,j}$ is the simulated absolute abundance. $N_{i,j}$ is the simulated observed count. The parameter of misclassification rate control level is denoted by η. $\mathcal{I}_1$ and $\mathcal{I}_2$ represent two different groups of samples defined by the differential abundant taxa.

Simulation study to assess compositional bias correction

The first experiment is conducted on the data set collected in He et al. (2018). We only include samples under the age of 30, so there are 539 samples and 37532 ASVs. The second experiment is conducted on the data set collected in Stämmler et al. (2016) containing 37 samples and 1783 OTUs.

Setting for Fig 2: In subfigure (a), (b), and (c), $n = 500$ samples are randomly selected from the data set and randomly divided into two groups $\mathcal{I}_1$ and $\mathcal{I}_2$ with equal size. 10% taxa are randomly selected to be $\mathcal{J}_1$. The idea of our simulation experiments is to treat the raw count data as the population of absolute abundance. Specifically, absolute abundance is generated in the following way:

$$\text{For } i \in \mathcal{I}_1: \qquad A_{i,j} = \begin{cases} X_{i,j} + \text{Poisson}(\lambda), & j \in \mathcal{J}_1, \\ X_{i,j}, \quad j \in \mathcal{J}_0 \end{cases}$$

$$\text{For } i \in \mathcal{I}_2: \qquad A_{i,j} = X_{i,j}, \quad j = 1, ..., d$$

Given the simulated absolute abundance, observed count in the simulation experiment is generated in the following way:

$$N_{i,j} \sim \text{Binomial}(A_{i,j}, c_i), \qquad i = 1, .., n, \quad j = 1, ..., d,$$

where $c_i \sim \text{Unif}[0, 1]$ is the sampling fraction of each sample. λ is 1 for the weak signal (a), 10 for the moderate signal (b), and 500 for the strong signal (c).

The setting for (d) is the same as (a), (b) and (c), except that we consider the proportion of differential abundant taxa as $p = 0.1, 0.2, 0.3$. The bias is evaluated in the following way:

$$\text{Bias} = \left| \frac{1}{|\mathcal{I}_1|} \sum_{i \in \mathcal{I}_1} \log\left(\hat{c}_i / c_i\right) - \frac{1}{|\mathcal{I}_2|} \sum_{i \in \mathcal{I}_2} \log\left(\hat{c}_i / c_i\right) \right|.$$

Here $| \cdot |$ represents the cardinality of a set. The two terms in above equation represent the average log difference between true and estimated sampling fraction for two groups respectively. If sampling fractions are correctly estimated for both two groups, the absolute

difference between these two terms should be close to zero. On the other hand, if the differential abundant taxa lead to a systematic bias in estimated sampling fraction, the bias can be large. In all above experiments, we choose $\eta = 0.2$.

Setting for Fig S4: The result of spike-in-based normalization serves as the ground truth for the sampling fraction, denoted as c_i for the ith sample. Let $\hat{c}_i$ denote the sampling fraction estimated by the computation-based normalization method. The discrepancy is evaluated in the following quantity:

$$\text{Discrepancy} = \left| \frac{1}{n} \sum_{i=1}^{n} \log(\hat{c}_i / c_i) \right|,$$

where n is the sample size.

Simulation study to assess the misclassification rate control

Similar to the last set of simulation experiments, we still conduct the experiments on data set collected in He et al. (2018). Each set of experiment is repeated 500 times and we use the average misclassification rate as measure.

Setting 1 for Fig S1a: $n = 500$ samples are randomly selected from the data set and divided into two groups $\mathcal{I}_1$ and $\mathcal{I}_2$ with equal size. 10% taxa are randomly selected to be $\mathcal{J}_1$. Absolute abundance is generated in the following way:

$$\text{For } i \in \mathcal{I}_1: \qquad A_{i,j} = \begin{cases} 10 \cdot \lambda \cdot (X_{i,j} + 1), & j \in \mathcal{J}_1 \\ 10 \cdot X_{i,j}, & j \in \mathcal{J}_0 \end{cases}$$

$$\text{For } i \in \mathcal{I}_2: \qquad A_{i,j} = 10 \cdot X_{i,j}, \quad j = 1, ..., d$$

λ is the signal strength and set to be 2 in this experiment. Observed count is generated in the following way:

$$N_{i,j} \sim \text{Binomial}(A_{i,j}, c_i), \qquad i = 1, .., n, \quad j = 1, ..., d,$$

where $c_i \sim \text{Unif}[0, 1]$ is the sampling fraction of each sample.

Setting 2 for Fig S1a: $n = 500$ samples are randomly selected from the data set and divided into two groups $\mathcal{I}_1$ and $\mathcal{I}_2$ with equal size. The top 10% abundant taxa are selected to be $\mathcal{J}_1$. Absolute abundance is generated in the following way:

$$\text{For } i \in \mathcal{I}_1: \qquad A_{i,j} = \begin{cases} \frac{10}{3} \cdot X_{i,j}, & j \in \mathcal{J}_1 \\ 10 \cdot X_{i,j}, & j \in \mathcal{J}_0 \end{cases}$$

$$\text{For } i \in \mathcal{I}_2: \qquad A_{i,j} = 10 \cdot X_{i,j}, \quad j = 1, ..., d$$

Observed count is generated in the following way:

$$N_{i,j} \sim \text{Binomial}(A_{i,j}, c_i), \qquad i = 1, .., n, \quad j = 1, ..., d,$$

where $c_i \sim \text{Unif}[0, 1]$ is the sampling fraction of each sample.

Setting 3 for Fig S1a: $n = 500$ samples are randomly selected from the data set. The top 10% abundant taxa are selected to be $\mathcal{J}_1$. First we generate a random variable $Y = [Y_1, .., Y_n]$ and Y_i's are drawn from Unif$[1, 100]$ independently. And absolute abundance is generated in the following way:

$$\text{For } i = 1, .., n : A_{i,j} = \begin{cases} 10 \cdot Y_i \cdot X_{i,j}, & j \in \mathcal{J}_1 \\ 10 \cdot X_{i,j}, & j \in \mathcal{J}_0 \end{cases}$$

Observed count is generated in the following way:

$$N_{i,j} \sim \text{Binomial}(A_{i,j}, c_i), \qquad i = 1, .., n, \quad j = 1, ..., d$$

where $c_i \sim \text{Unif}[0, 1]$ is the sampling fraction of each sample.

Setting for Fig S1b: Three settings are the same as Fig S1a. We set $\eta = 0.1$ and change γ from 0.5 to 0.95.

Setting for Fig S2a and S2b: The setting of power comparison is the same as Fig S7a with $n = 500$, the setting of type I error comparison is the same as Fig 4a with $c = 3$.

Setting for Fig S2c and S2d: The setting is the same as Fig 5. For Pearson correlation test, we choose $n = 200$ and $\lambda = 50$. For t-test, we choose $n = 100$ and $\lambda = 50$.

Setting for Fig S3: All the four subfigures share the same data structure with the first setting of Fig S1a. Signal strength λ increases from 1 to 10; the ratio between the group sizes increases from 0.1 to 0.4; the proportion of differential abundant taxa increases from 0.1 to 0.5; sample size increases from 100 to 500. We choose $\eta = 0.01$ in the experiments.

Simulation study for PCoA

The Euclidean distance is used in all the PCoA plots.

Setting for Fig 3a and Fig 3b: The experiment is conducted on data set collected in He et al. (2018). We only include samples with total counts larger than 30000 in this experiment. Fig 3a is the result for raw data. For 3b, half of the samples are subsampled to 1/10 of the original sequencing depth, while the other half of the samples remain the same. $\eta = 0.2$ in these experiments.

Setting for Fig 3c: PCoA performance on the data set collected in Vangay et al. (2018). The setup of this experiment can be found in PCoA section.

Setting for Fig S5a: All the samples of the data set in He et al. (2018) are included in this experiment. Samples are divided into two groups $\mathcal{I}_1$ and $\mathcal{I}_2$. The top 25% abundant taxa are selected to be $\mathcal{J}_1$. Absolute abundance is generated in the following way:

$$\text{For } i \in \mathcal{I}_1 : \qquad A_{i,j} = \begin{cases} 1000 \cdot X_{i,j}, & j \in \mathcal{J}_1 \\ 20 \cdot X_{i,j}, & j \in \mathcal{J}_0 \end{cases}$$

$$\text{For } i \in \mathcal{I}_2 : \qquad A_{i,j} = 20 \cdot X_{i,j}, \quad j = 1, ..., d$$

Observed count is generated in the following way:

$$\begin{aligned} \text{For } i \in \mathcal{I}_1 : \qquad \log(c_i) &\sim \text{Normal}(-3, 0.25) \\ N_{i,j} &\sim \text{Binomial}(A_{i,j}, \min(c_i, 1)), \qquad j = 1, .., d \\ \text{For } i \in \mathcal{I}_2 : \qquad \log(d_i) &\sim \text{Normal}(0, 0.25) \\ N_{i,j} &\sim \text{Binomial}(A_{i,j}, \min(d_i, 1)), \qquad j = 1, .., d \end{aligned}$$

The sampling fraction of $\mathcal{I}_1$ is around ten times of $\mathcal{I}_2$.

Setting for Fig S5b: All the samples are included in this experiment($n = 539$). The top 1% abundant taxa are selected to be $\mathcal{J}_1$. First we generate a random variable $Y = [Y_1, .., Y_n]$ and Y_i's are drawn from Unif$[5, 500]$ independently. Absolute abundance is generated in the following way:

$$\text{For } i = 1, .., n : \qquad A_{i,j} = \begin{cases} Y_i \cdot X_{i,j}, & j \in \mathcal{J}_1 \\ X_{i,j}, & j \notin \mathcal{J}_1 \end{cases}$$

Observed abundance is generated in the following way:

$$N_{i,j} \sim \text{Binomial}(A_{i,j}, \min(c_i, 1)), \qquad i = 1, .., n, \quad j = 1, ..., d$$

where $1/c_i \sim N(Y_i, 1)$ and $\min(c_i, 1)$ is the sampling fraction for sample i.

Simulation study for association analysis

Simulation experiments is still based on the data set in He et al. (2018). All the experiments for the association analysis are repeated 500 times.

Setting for Fig 4a: $n = 500$ samples are divided into two groups with equal size. Samples in the first group are subsampled to $1/c$ of the original sequencing depth, while the second group keeps the same as the original data set. c increases from 1 to 3. We choose $\eta = 0.07$.

Setting for Fig 4b: We consider sample size $n = 100, 300, 500$. Samples are divided into two groups $\mathcal{I}_1$ and $\mathcal{I}_2$ with equal size. The top 10% abundant taxa are selected to be $\mathcal{J}_1$. Absolute abundance is generated in the following way:

$$\text{For } i \in \mathcal{I}_1: \qquad A_{i,j} = \begin{cases} \lambda \cdot X_{i,j}, & j \in \mathcal{J}_1 \\ X_{i,j}, & j \in \mathcal{J}_0 \end{cases}$$
$$\text{For } i \in \mathcal{I}_2: \qquad A_{i,j} = X_{i,j} \quad j = 1, ..., d.$$

Here we choose $\lambda = 2$. Observed count is generated in the following way:

$$\begin{aligned} \text{For } i \in \mathcal{I}_1: \qquad \log(c_i) &\sim \text{Normal}(-0.7, 0.05) \\ N_{i,j} &\sim \text{Binomial}(A_{i,j}, \min(c_i, 1)), \qquad j = 1, .., d \\ \text{For } i \in \mathcal{I}_2: \qquad \log(d_i) &\sim \text{Normal}(0, 0.05) \\ N_{i,j} &\sim \text{Binomial}(A_{i,j}, \min(d_i, 1)), \qquad j = 1, .., d \end{aligned}$$

The sampling fraction of $\mathcal{I}_1$ is around two times of $\mathcal{I}_2$. We choose $\eta = 0.05$.

Setting for Fig S7a: The setting of Fig S7a is the same as Fig 4b except that $\lambda = 5$.

Setting for Fig S7b: We consider sample size $n = 100, 300, 500$. The setting is the same with Fig S1c.

Simulation study for differential abundance test

There are three parts in this section. The first set of experiments is based on the data set in He et al. (2018), of which results are shown in Fig 5; the second set is based on the data set in Caporaso et al. (2011), of which result is shown on Fig 6; the third set is based on the data set in Vangay et al. (2018), of which result is shown on Table S1. We use Benjamini-Hochberg procedure to adjust the effect of multiple testing.

For the first set of the simulation study, to reduce the computational load, we select the subtree below Node 351 as our target, which contains 1081 taxa in total. We repeat each experiment 500 times and consider the average sensitivity rates and false discovery rates.

Setting for Fig 5a and 5b: We consider sample size $n = 100, 200, 300$. Samples are divided into two groups $\mathcal{I}_1$ and $\mathcal{I}_2$ with equal size. 10% taxa are randomly selected to be $\mathcal{J}_1$. Absolute abundance is generated in the following way:

$$\text{For } i \in \mathcal{I}_1: \qquad A_{i,j} = \begin{cases} \lambda \cdot (X_{i,j} + \text{Poisson}(\lambda)), & j \in \mathcal{J}_1 \\ \lambda \cdot X_{i,j}, & j \in \mathcal{J}_0 \end{cases}$$
$$\text{For } i \in \mathcal{I}_2: \qquad A_{i,j} = \lambda \cdot X_{i,j}, \quad j = 1, ..., d.$$

Observed count is generated in the following way:

$$\begin{aligned}
\text{For } i \in \mathcal{I}_1: \quad & \log(c_i) \sim \text{Normal}(-\log(\lambda), 0.01) \\
& N_{i,j} \sim \text{Binomial}(A_{i,j}, \min(c_i, 1)), \qquad j = 1, .., d \\
\text{For } i \in \mathcal{I}_2: \quad & \log(d_i) \sim \text{Normal}(-\log(\lambda/10), 0.01) \\
& N_{i,j} \sim \text{Binomial}(A_{i,j}, \min(d_i, 1)), \qquad j = 1, .., d
\end{aligned}.$$

λ is chosen to be 50,100,150. Two-sample t-test is applied on the normalized counts. The significance level is 0.1. We choose $\eta = 0.01$.

Setting for Fig 5c and 5d: We consider sample size $n = 200, 350, 500$. The top 10% abundant taxa are selected as $\mathcal{J}_1$. First we generate a random vector $Y = [Y_1, .., Y_n]$ and Y_i are draw from Unif$[1, \lambda]$. The absolute abundance is generated in the following way:

$$\text{For } i = 1, .., n: \qquad A_{i,j} = \begin{cases} X_{i,j} + \text{Poisson}(Y_i), & j \in \mathcal{J}_1 \\ X_{i,j}, & j \in \mathcal{J}_0 \end{cases}$$

Then we generate the sampling fraction in the following way:

$$c_i \sim \begin{cases} \text{Unif}[0.5, 1], & Y_i > \text{mean}(Y_i) \\ \text{Unif}[0.1, 0.5], & Y_i \leq \text{mean}(Y_i) \end{cases}$$

Observed abundance is generated in the following way:

$$N_{i,j} \sim \text{Binomial}(A_{i,j}, c_i), \qquad i = 1, .., n, \quad j = 1, ..., d$$

λ is chosen to be 50, 100, and 150. Pearson correlation test is applied on normalized counts. The significance level is 0.1 and we choose $\eta = 0.01$.

Setting for Fig 6: Samples of subject M3's right palm are selected from data set in Caporaso et al. (2011). In total there are 352 samples and 24333 ASVs. Samples with a sequencing depth of less than 10000 are selected to be the first group, and samples with a sequencing depth of more than 20000 are another group. ANCOM, EdgeR, LinDA, RDB and t-test with different normalization methods are considered in the experiment. We use the detected_0.9 for ANCOM, which is the most conservative setting, and the default setting for EdgeR, RDB and LinDA.

Setting for Table S1: Differential abundant test is conducted for group KareThai and group Karen1st from data set in Vangay et al. (2018). For t-test with RSim, data is first normalized at the ASV level, then aggregated to the phylum level. The FDR control level is 0.1.

References

J. G. Caporaso, C. L. Lauber, E. K. Costello, D. Berg-Lyons, A. Gonzalez, J. Stombaugh, D. Knights, P. Gajer, J. Ravel, N. Fierer, J. I. Gordon, and R. Knight. Moving pictures of the human microbiome. *Genome Biology*, 12(5):1–8, 2011.

Y. He, W. Wu, H. Zheng, P. Li, D. McDonald, H. Sheng, M. Chen, Z. Chen, G. Ji, Z. Zheng, P. Mujagond, X. Chen, Z. Rong, P. Chen, L. Lyu, X. Wang, C. Wu, N. Yu, Y. Xu, J. Yin, J. Raes, R. Knight, W. Ma, and H. Zhou. Regional variation limits applications of healthy gut microbiome reference ranges and disease models. *Nature Medicine*, 24(10):1532–1535, 2018.

F. Stämmler, J. Gläsner, A. Hiergeist, E. Holler, D. Weber, P. J. Oefner, A. Gessner, and R. Spang. Adjusting microbiome profiles for differences in microbial load by spike-in bacteria. *Microbiome*, 4:1–13, 2016.

P. Vangay, A. J. Johnson, T. L. Ward, G. A. Al-Ghalith, R. R. Shields-Cutler, B. M. Hillmann, S. K. Lucas, L. K. Beura, E. A. Thompson, L. M. Till, R. Batres, B. Paw, S.L. Pergament, P. Saenyakul, M. Xiong, A. D. Kim, G. Kim, D. Masopust, E. C. Martens, C. Angkurawaranon, R. McGready, P. C. Kashyap, K. A. Culhane-Pera, and D. Knights. Us immigration westernizes the human gut microbiome. *Cell*, 175(4):962–972, 2018.
