# Supplementary figures and images for: RSim: A reference-based normalization method via rank similarity
Source: PLoS Comput Biol. 2023 Sep 1;19(9):e1011447. doi: 10.1371/journal.pcbi.1011447 (PMC10501661; doi:10.1371/journal.pcbi.1011447)

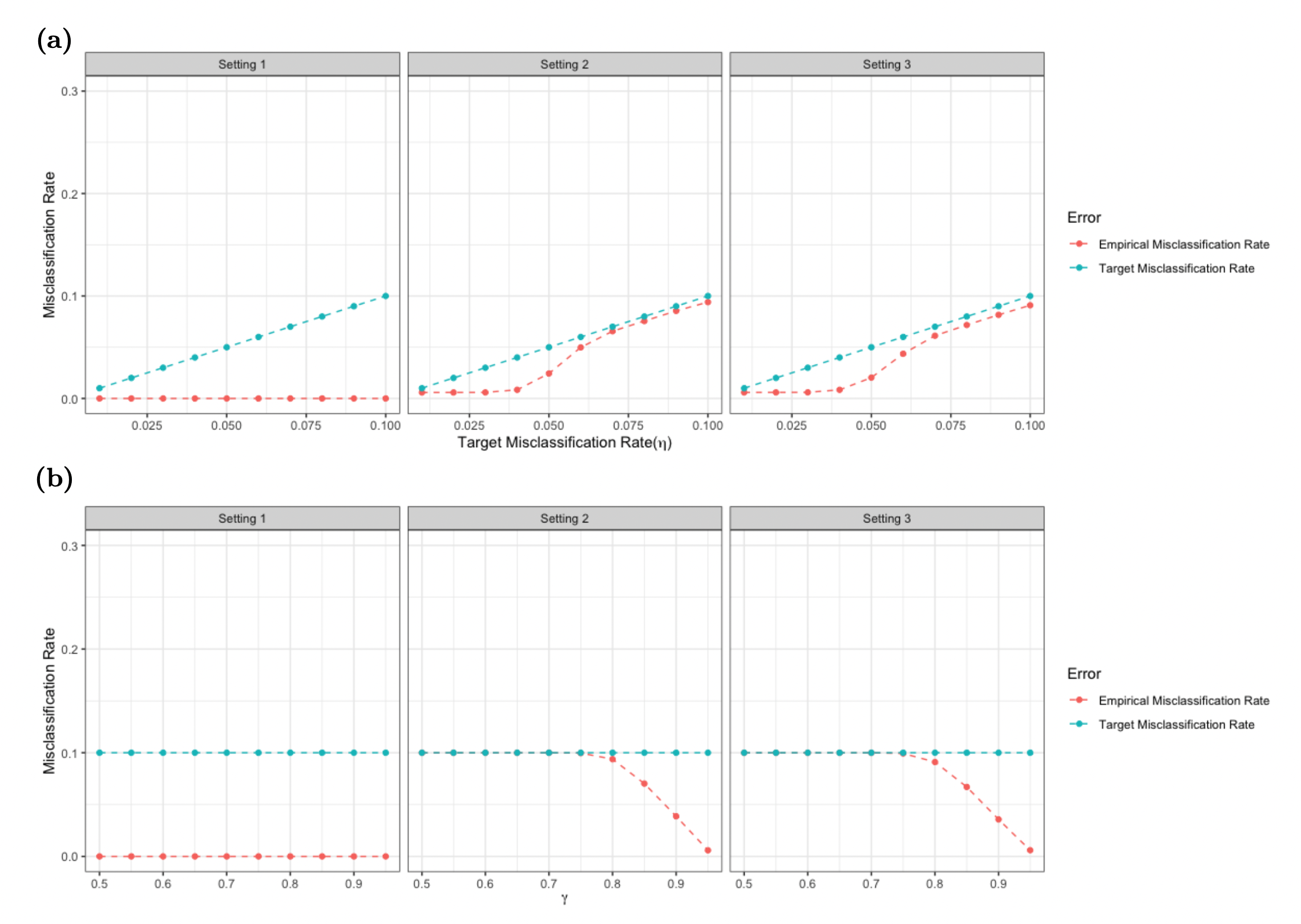

Supplement: S1 Fig — In Fig (a), the x-axis is the target misclassification rate, while the y-axis represents the empirical misclassification rate of the estimated reference set. In all settings, the misclassification error rate of the estimated reference set can be well controlled. In Fig (b), we vary the value of γ from 0.5 to 0.95. All three settings are the same for both figures. Setting 1: 10% taxa are randomly selected as differential abundant taxa, and the latent variable of differential abundant taxa is binary; Setting 2: the differential abundant taxa are top 10% most abundant taxa, and the latent variable of differential abundant taxa is binary; Setting 3: the differential abundant taxa are top 10% most abundant taxa, and the latent variable of differential abundant taxa is continuous. (PNG) [file pcbi.1011447.s002.png]

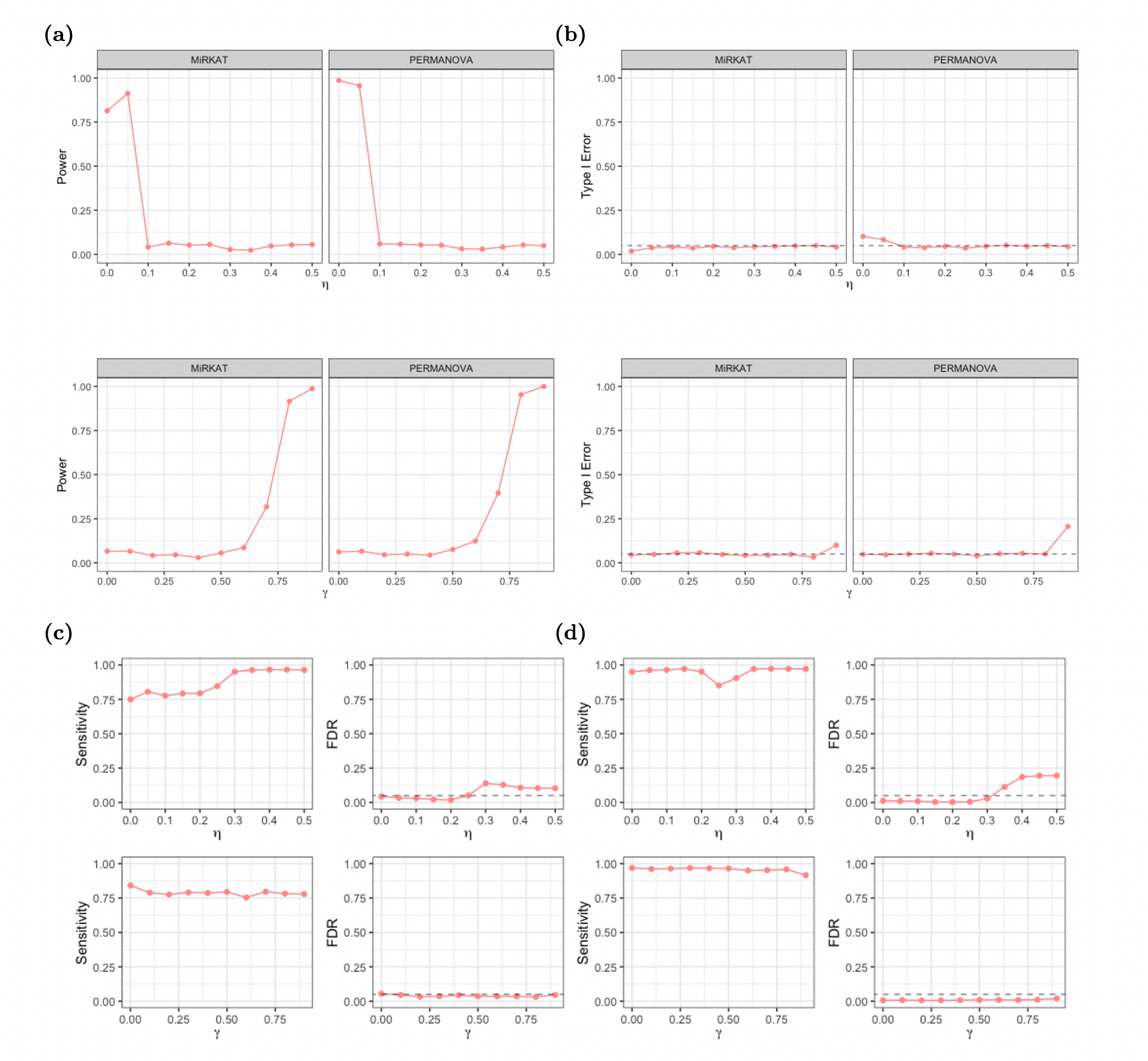

Supplement: S2 Fig — (a) and (b) show how the tuning parameter choices influence association analysis. Small η and large γ will lead to a higher power. (c) and (d) show how the tuning parameter choices will influence the Pearson correlation test and t-test, respectively. FDR of Pearson correlation test and t-test results has inflation if η value is large. (PNG) [file pcbi.1011447.s003.png]

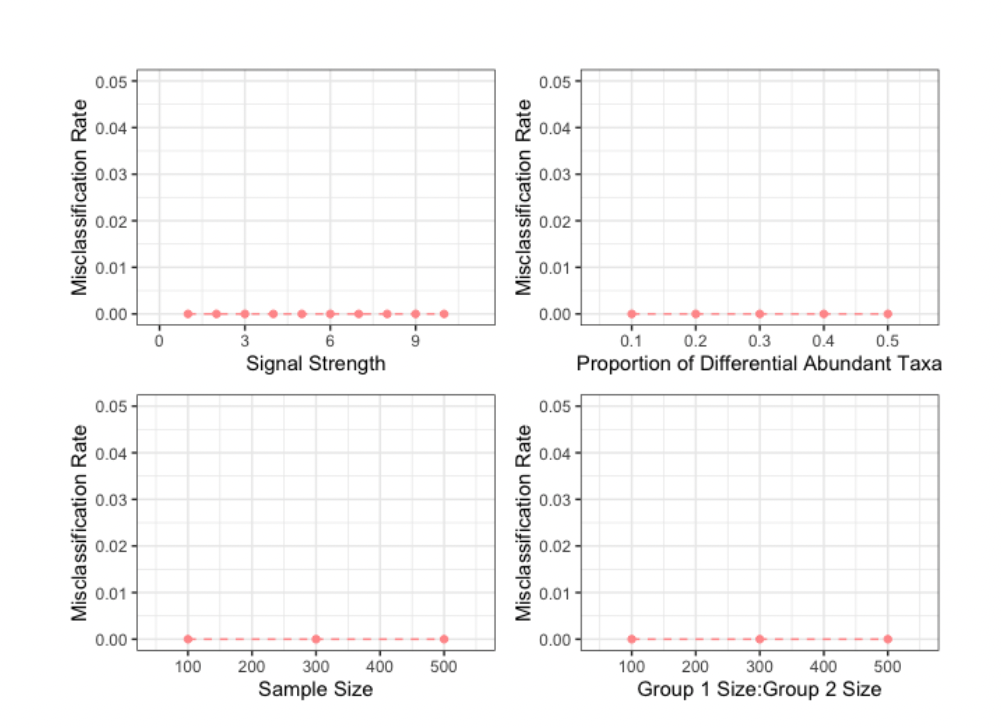

Supplement: S3 Fig — The empirical misclassification rate in RSim is well controlled despite the choices of the signal strength of differential abundant taxa, the balance of group size, proportion of differential abundant taxa, and sample size. (PNG) [file pcbi.1011447.s004.png]

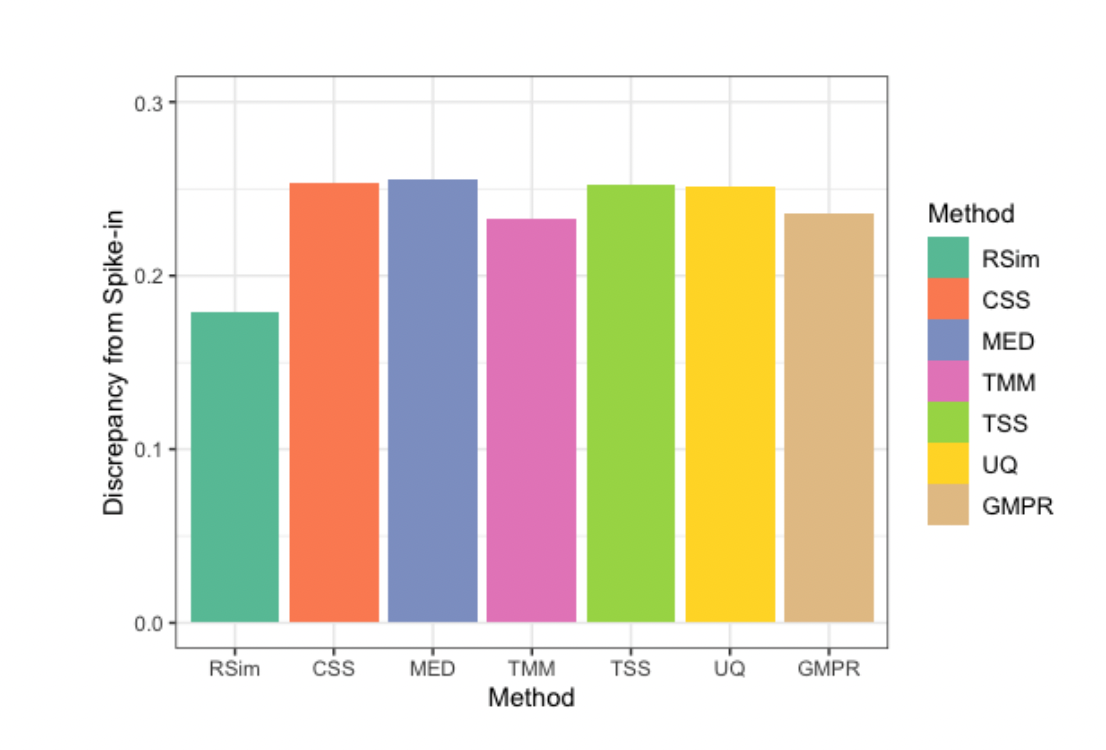

Supplement: S4 Fig — The discrepancies from the spike-in-based normalization method are compared, and it is observed that RSim exhibited the closest results to the spike-in-based normalization method. (PNG) [file pcbi.1011447.s005.png]

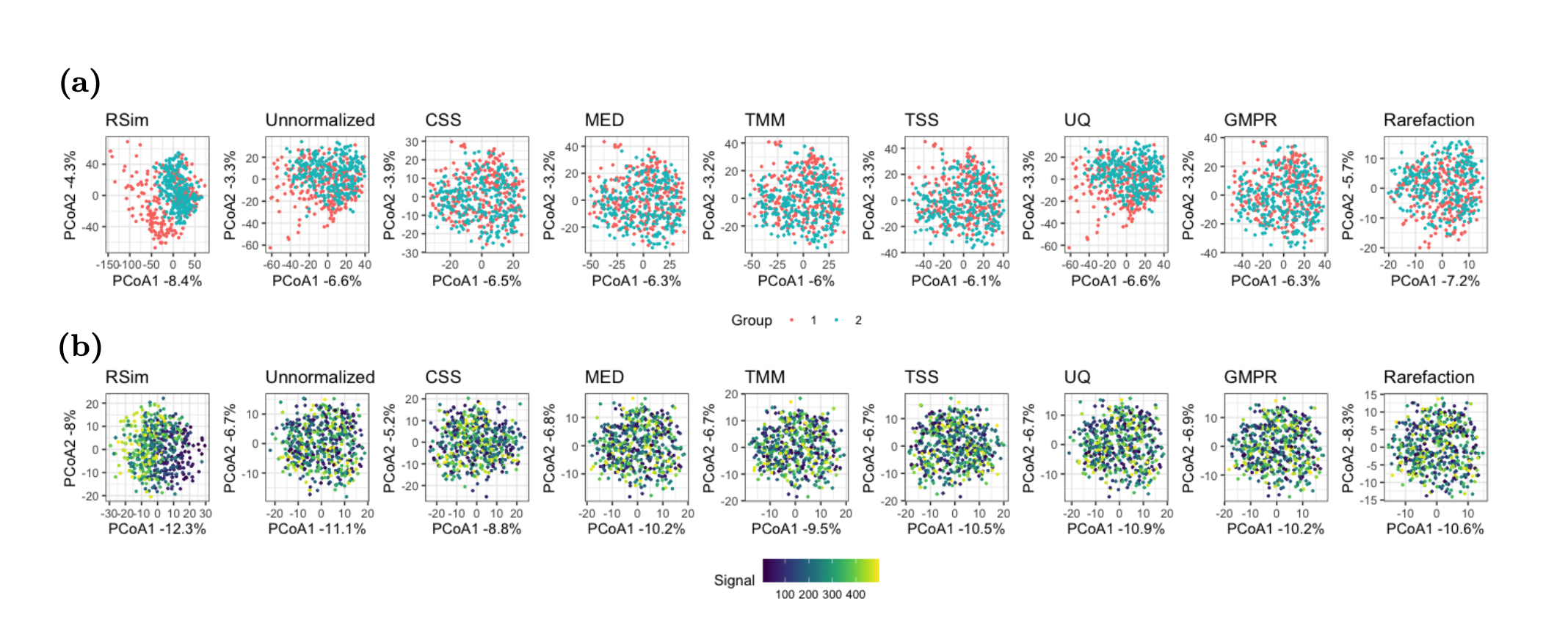

Supplement: S5 Fig — In (a), samples are randomly divided into two groups, and the top 10% most abundant taxa are differential abundant taxa with a binary latent variable. In (b), the top 10% most abundant taxa are differential abundant taxa with a continuous latent variable. In these figures, RSim normalization can reveal the structure of the latent variable. Euclidean distance with log transformation is used in all PCoA plots. (PNG) [file pcbi.1011447.s006.png]

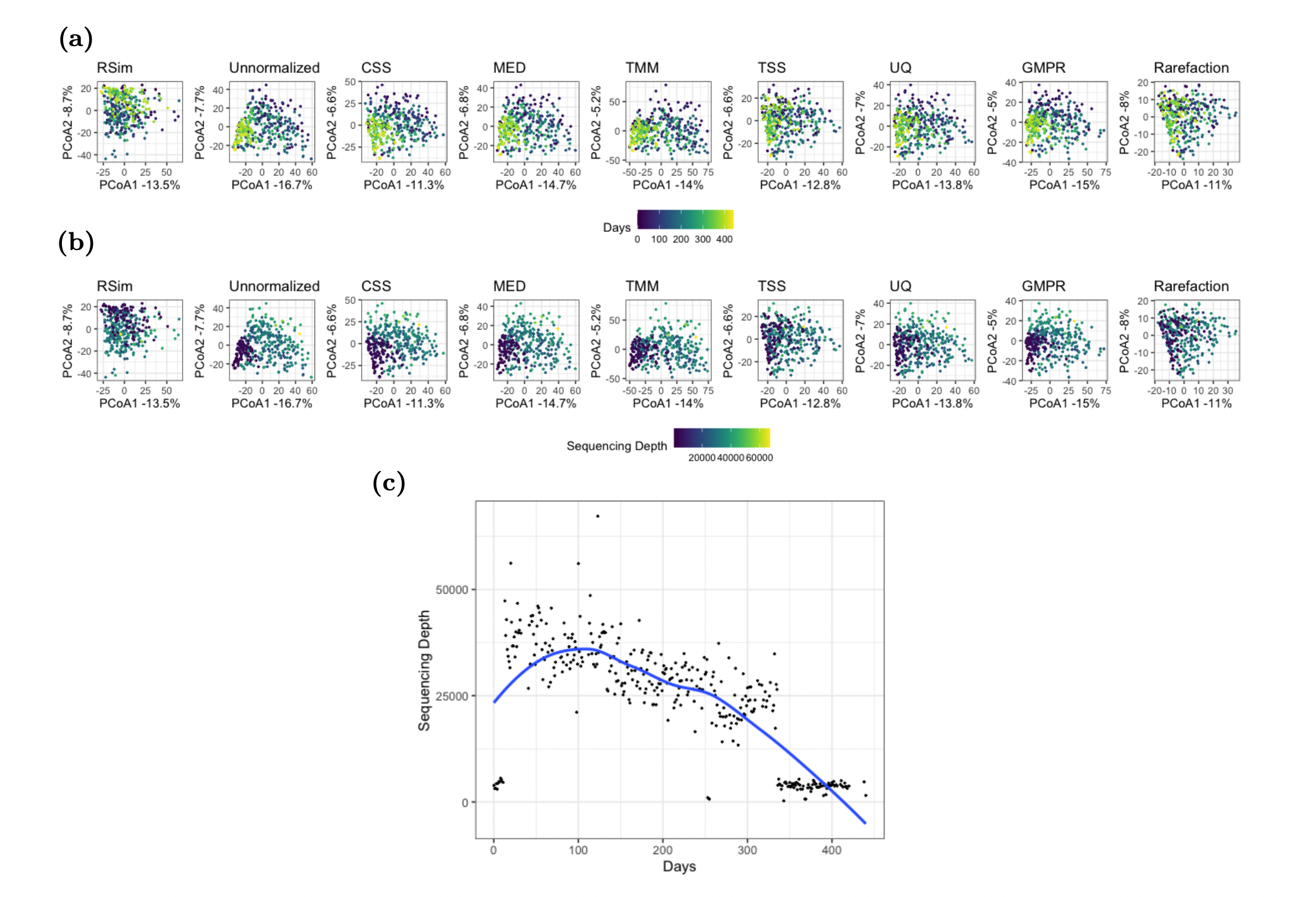

Supplement: S6 Fig — (a) shows the PCoA plots colored by days after the experiment started. (b) presents the PCoA plots colored by sequencing depth. (c) show the relationship between time and sequencing depth. The pattern of time in PCoA plots is highly overlapped with pattern of the sequencing depth, which can be explained by the deterministic relationship between time and sequencing depth. (PNG) [file pcbi.1011447.s007.png]

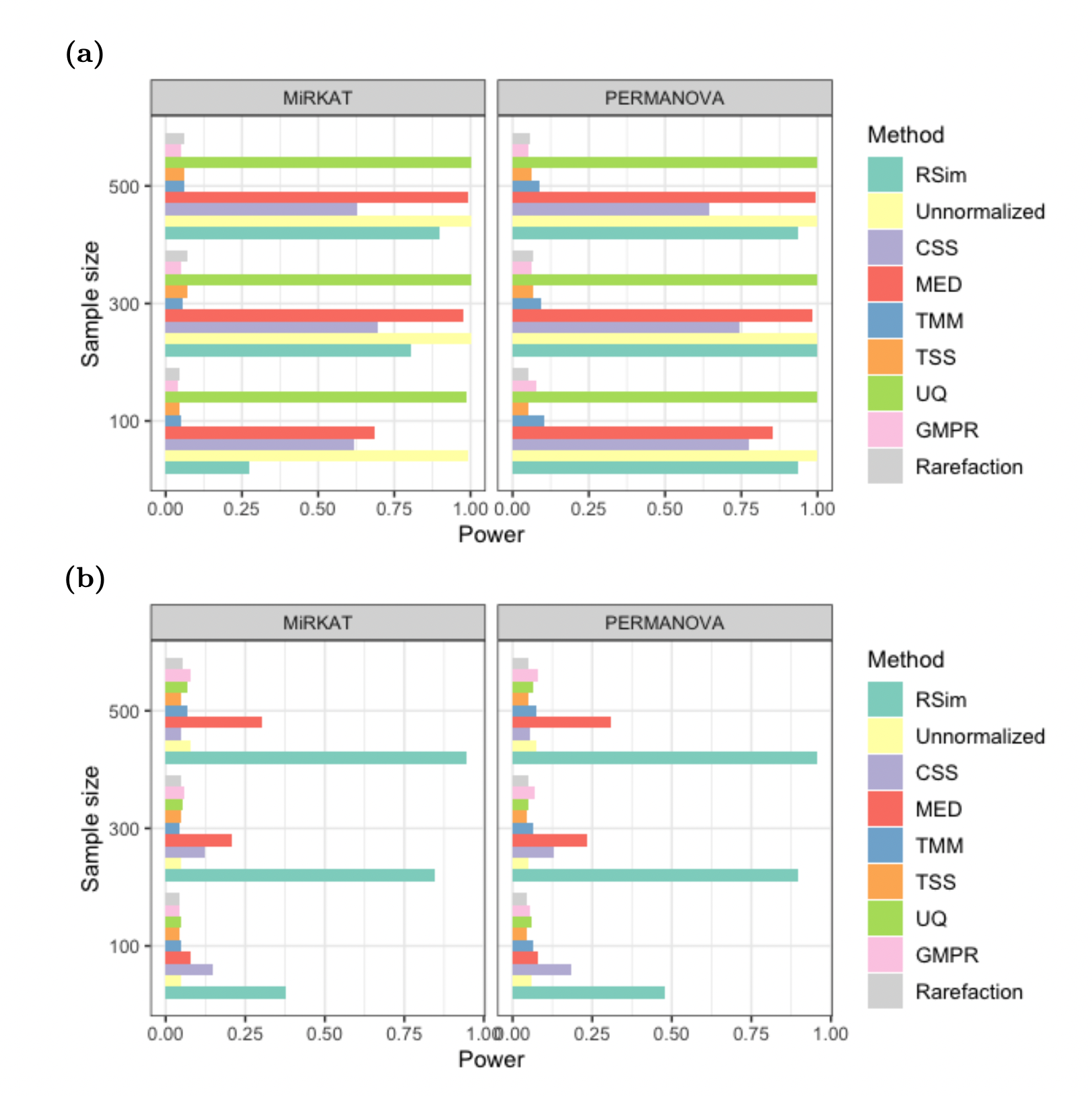

Supplement: S7 Fig — In (a) and (b), samples are randomly divided into two groups, and the top 25% most abundant taxa are differential abundant taxa with a binary or continuous latent variable. The significance level is 0.05. RSim can improve the power of association analysis. (PNG) [file pcbi.1011447.s008.png]
