# Supplementary material for: RSim: A reference-based normalization method via rank similarity
Source: PLoS Comput Biol. 2023 Sep 1;19(9):e1011447. doi: 10.1371/journal.pcbi.1011447 (PMC10501661; doi:10.1371/journal.pcbi.1011447)
Supplement: S1 Table — Three methods are considered: t-test on unnormalized data, t-test on data normalized by RSim, and RDB test on unnormalized data. (PDF) [file pcbi.1011447.s009.pdf]

Method	Phylum
t-test on unnormalized data	Cyanobacteria<br>Elusimicrobiota<br>Proteobacteria
t-test on data normalized by RSim	Actinobacteriota<br>Bacteroidota<br>Desulfobacterota<br>Firmicutes<br>Fusobacteriota<br>Patescibacteria
RDB test on unnormalized data	Actinobacteriota<br>Bacteroidota<br>Desulfobacterota<br>Firmicutes<br>Fusobacteriota<br>Patescibacteria
