# Supplementary material for: RSim: A reference-based normalization method via rank similarity
Source: PLoS Comput Biol. 2023 Sep 1;19(9):e1011447. doi: 10.1371/journal.pcbi.1011447 (PMC10501661; doi:10.1371/journal.pcbi.1011447)
Supplement: S2 Table — d is the number of taxa, n is the sample size. All the experiments are conducted in iMac M1/8GB. Data are subsampled from the dataset collected in [30]. (PDF) [file pcbi.1011447.s010.pdf]

$d \times n$	RSim	GMPR	MED	CSS	TMM	TSS	UQ
1000×100	0.296	0.487	0.013	0.136	0.045	0.013	0.022
5000×100	4.702	1.886	0.043	0.239	0.111	0.064	0.076
10000×100	17.069	3.455	0.077	0.336	0.178	0.123	0.146
1000×500	0.562	10.907	0.069	0.657	0.221	0.068	0.115
5000×500	12.406	47.221	0.222	1.243	0.693	0.323	0.399
10000×500	48.891	87.821	0.416	2.026	1.201	0.657	0.754
